# SEQUENTIAL ANALYSIS OF DEMENTIA FAMILY DYADIC COMMUNICATION: EFFECT OF CAREGIVER DISABLING COMMUNICATION

**DOI:** 10.1093/geroni/igad104.3586

**Published:** 2023-12-21

**Authors:** Sohyun Kim, Will Chen, Shan Sun-Mitchell

**Affiliations:** University of Texas at Arlington, Arlington, Texas, United States; University of Texas at Arlington, Arlington, Texas, United States; University of Texas at Arlington, Arlington, Texas, United States

## Abstract

This study examined temporal relationships between antecedent family caregiver disabling communication and subsequent care recipient (persons living with dementia) communication during 75 in-home care video observations. Secondary analysis of 5-, 10-, 15-, 20-, 25-, and 30-second timed-window analysis was conducted. 95% confidence intervals, p-values, and Yule’s Q statistics were used. The results showed that care recipient engaging, challenging, and neutral communication occurred at differing frequencies within each time window following disabling communication by family caregivers. Care recipient challenging verbal communication was more likely to occur within all time widows after caregiver challenging verbal communication preceded (OR = 1.51 – 1.77, p <.001, Yule’s Q = 0.204 – 0.277). Care recipient engaging verbal communication was more likely to occur with weaker chance within all time widows except 30-second window after caregiver disabling verbal communication preceded (OR = 1.11 – 1.17, p = .001 - .028, Yule’s Q = 0.151 – 0.080). Care recipient neutral communication was more likely to occur within all time widows after caregiver disabling verbal communication preceded (OR = 1.34 – 1.39, p <.001, Yule’s Q = 0.144 – 0.162). Care recipient neutral communication more likely occurred within all time widows except 5-second and 10-second windows after caregiver disabling nonverbal communication preceded (OR = 1.28 – 1.34, p =.009 - .029, Yule’s Q = 0.123 – 0.144). Caregiver disabling communication was associated with more likely subsequent care recipient communication. Future research can further explore how participant characteristics and types and frequencies of caregiver communication influence care recipient communication.

